# Serum and Urine Neutrophil Gelatinase-Associated Lipocalin Levels Measured at Admission Predict Progression to Chronic Kidney Disease in Sepsis-Associated Acute Kidney Injury Patients

**DOI:** 10.1155/2020/8883404

**Published:** 2020-08-17

**Authors:** Pham Ngoc Huy Tuan, Dao Bui Quy Quyen, Huynh Van Khoa, Nguyen Duc Loc, Pham Van My, Nguyen Huu Dung, Nguyen Duy Toan, Do Quyet, Le Viet Thang

**Affiliations:** ^1^Trung Vuong Hospital, Ho Chi Minh, Vietnam; ^2^Cho Ray Hospital, Ho Chi Minh, Vietnam; ^3^An Sinh Hospital, Ho Chi Minh, Vietnam; ^4^University of Medicine Pham Ngoc Thach, Ho Chi Minh, Vietnam; ^5^Bach Mai Hospital, Ha Noi, Vietnam; ^6^Military Hospital 103, Ha Noi, Vietnam; ^7^Vietnam Military Medical University, Ha Noi, Vietnam

## Abstract

**Background:**

To evaluate the ratio of acute kidney injury (AKI) to chronic kidney disease (CKD) in sepsis-associated acute kidney injury (SA-AKI) patients of the intensive care unit (ICU) and predictive value of neutrophil gelatinase-associated lipocalin (NGAL) measured at the admission time in the progression of AKI to CKD.

**Methods:**

A study of 121 consecutive adult patients admitted to the intensive care unit (ICU) diagnosed as SA-AKI. AKI and CKD were defined based on Kidney Disease: Improving Global Outcomes (KDIGO) criteria. Glomerular filtration rate (GFR) was calculated by the CKD-EPI formula. Serum and urine NGAL was measured using the BioVendor Human Lipocalin-2/NGAL ELISA with a blood sample taken at hospital admission time.

**Results:**

The ratio of AKI to CKD in SA-AKI patients was 22.3%. Mean concentration of serum and urine NGAL in AKI to the CKD group was 790.99 ng/ml and 885.72 ng/ml, higher significantly than those of recovery patients (351.86 ng/ml and 264.68 ng/ml), *p* < 0.001. eGFR, both serum and urine NGAL had a predictive value for AKI to CKD (eGFR: AUC = 0.857, Se = 74.1%, Spe = 92.6%, *p* < 0.001. Serum NGAL: AUC = 0.868, Se = 77.8%, Spe = 91.5%. Urine NGAL: AUC = 0.869, Se = 77.8%, Spe = 92.6%, *p* < 0.001.

**Conclusion:**

Serum and urine NGAL, measuring at hospital admission time, were good prognostic biomarkers of AKI to CKD in SA-AKI patients.

## 1. Introduction

Acute kidney injury (AKI) affects 20 to 50% of intensive care unit (ICU) patients, and it is associated with high mortality and increased ICU length of stay [1–3]. Sepsis was the most common cause of AKI [3–6]. The role of intrarenal and systemic inflammation appears to be significant in the pathophysiology of sepsis-associated acute kidney injury (SA-AKI) [7]. Sepsis-associated AKI patients have distinct characteristics than non-SA-AKI, that is, higher severity scores at admission, more nonrenal organ failure, and requirement of vasopressors and mechanical ventilation [8, 9]. Rising serum creatinine levels from a basal level are considered as the gold standard for the diagnosis of impaired renal function in the current consensus definitions [10]. Recently, serum and urine neutrophil gelatinase-associated lipocalin (NGAL) levels were used as a biomarker to early diagnosis of AKI in both SA-AKI and non-SA-AKI patients [11–13]. Serum and urine NGAL levels were also used to predict mortality in SA-AKI patients [14, 15].

AKI is associated with high mortality, ranging from 25% to nearly 60% depending on severity as well as SA-AKI [9, 16]. Among survivors, the long-term consequences of AKI include the development of end-stage renal disease (ESRD) and chronic kidney disease (CKD). Several clinical studies have also reported that even mild AKI with apparent complete recovery might lead to the subsequent development of CKD [17, 18]. Some of the important insights into AKI to CKD progression are that aging and inflammation are relevant risk factors for CKD progression after AKI [19–21]. In this study, we assume that serum and urine NGAL can predict the progression of AKI to CKD in SA-AKI patients.

## 2. Materials and Methods

### 2.1. Study Design and Setting

We studied prospectively 121 adult patients diagnosed as SA-AKI admitted to the ICU, Trung Vuong Hospital, Ho Chi Minh, Viet Nam. We excluded patients below 16 years of age, patients with non-SA-AKI, patients with chronic kidney disease, organ transplant recipients, patients with malignancy, pregnant women, patients admitted for observation to stay in the ICU for <48 h, and died patients with all-cause. To determine the predictive value of both urine and serum NGAL, all patients with anuria were excluded from the study. A written informed consent and Hospital's Ethics Committee clearance were obtained before the recruitment of the participants for the study.

Preexisting comorbidities such as diabetes mellitus and hypertension and medications were noted. Diabetes mellitus was identified according to either a physician's diagnosis, antidiabetic drug treatment, or 2 subsequent analyses demonstrating fasting blood glucose levels of >126 mg/dl or >7.0 mmol/l. Hypertension was defined as the regular use of antihypertensive drugs for controlling blood pressure or at least 2 blood pressure measurements of >140/90 mm Hg. Demographic data such as age, gender, comorbid conditions, clinical setting such as medical or surgical, and presence of sepsis were noted. In our study, we used the Sequential Organ Failure Assessment (SOFA) score to define single organ failure (SOF) and multiple organ failure (MOF) [22]. SOF was defined when the SOFA score was more than 3 in a particular organ at any time of hospitalization [23]. MOF was defined when two or more organ failure was confirmed (Table 1).

### 2.2. Definitions

Acute kidney injury was defined based on Kidney Disease: Improving Global Outcomes (KDIGO) criteria [24]. Patients were classified as the severity of AKI was graded as (1) Stage 1 in proportion to a 1.5-1.9-fold increasing of the SCr level or an acute rise in SCr of more than 26.5 *μ*mol/l (0.3 mg/dl) within 48 h, (2) Stage 2 in proportion to a 2.0-2.9-fold increasing of the SCr level, and (3) Stage 3 in proportion to a 3.0-fold increasing of SCr or SCr ≥ 353.6 *μ*mol/l (4.0 mg/dl) or need for dialysis.

Sepsis in our study was defined as having manifestations of systemic inflammatory response syndrome (SIRS) in combination with evidence of infection in the blood (expressed by positive bacterial cultures). SIRS was defined as having two or more of [25] temperature > 38°C or <36°C, heart rate > 90/min, respiratory rate > 20/min or PaCO_2_ < 32 mm Hg (4.3 kPa), white blood cell count > 12, 000/mm^3^ or <4000/mm^3^, or >10% immature bands. SA-AKI was defined by AKI in the presence of sepsis without another significant contributing factor that might cause AKI, such as nephrotoxic drugs, poisoning, bleeding, and hypovolemic circulatory.

### 2.3. Laboratory Measurements

Blood urea, SCr, serum electrolytes, and other biochemical parameters of organ function were measured at admission. SCr was measured at admission time and daily thereafter. The steady level of creatinine at 4 weeks before admission was estimated to define baseline SCr. Otherwise, the admission value or the lowest SCr during hospitalization was used as a surrogate baseline. Serum NGAL was measured by the BioVendor Human Lipocalin-2/NGAL ELISA kit based on the sandwich enzyme immunoassay method. All patients were calculated glomerular filtration rate (eGFR) basing on the CKD-EPI formula at MDRD.COM.

At the time of admission, the patient was given a catheter to collect urine for 24 hours. After 24 hours, measure the volume of urine, take 5 ml of urine to determine the NGAL level, then calculate the 24-hour urine NGAL concentration. Oliguria was defined as urine 24-hour volume from 240 to <500 ml; anuria was determined as urine 24-hour volume < 240 ml (<10 ml/hour). Urine NGAL was measured by the BioVendor Human Lipocalin-2/NGAL ELISA kit based on the sandwich enzyme immunoassay method.

### 2.4. Study Outcomes

All patients received standard routine care and get treatment as the guideline of Viet Nam Health Ministry as well as KDIGO 2012 [24]. The first outcome that we defined was the incidence of SA-AKI occurring during ICU stay, the second outcome was the recovery and hospital mortality during patients' hospital stay, and the final outcome was the progression of AKI to CKD in SA-AKI patients after 90 days.

After discharging from the hospital, all patients were reviewed every month. Urine analysis, blood tests for full blood count, serum urea, creatinine, eGFR calculation, and kidney ultrasound have been done in all patients.

CKD was defined as kidney damage for ≥3 months (90 days) with either [26] of the following: (1) structural or functional abnormalities of the kidney, with or without decreased glomerular filtration rate (GFR) as seen by either (a) pathological abnormalities or (b) markers of kidney damage, including abnormalities in the blood or urine, or abnormalities seen on imaging OR; (2) GFR < 60 ml/min/1.73 m^2^, with or without kidney damage.

### 2.5. Statistical Analyses

All the normal distribution, continuous data were represented by mean and standard deviation and were analyzed by the Student *t*-test, one-way ANOVA, and post hoc Bonferroni test. All the skewed distributions were represented by median (25 percentile–75 percentile), analyzed by the Mann-Whitney *U* test and Kruskal-Wallis test. Categorical data were presented by frequency with percentage and were analyzed using the chi-square test. Multivariable adjusted regression analysis was performed to identify the predictors of the progression of AKI to CKD. Receiver operating characteristic (ROC) curves with the area under the curve (AUC) was calculated to predict the progression of AKI to CKD from all patients. Statistical analysis was done using Statistical Package for Social Science (SPSS) version 20.0 (Chicago, IL, USA). A *p* value < 0.05 was considered as significant.

## 3. Results

The baseline demographic and laboratory characteristics in SA-AKI patients are shown in Table 2. The mean age of SA-AKI patients was 67.81 ± 16.04 years old, 59.5% were male, 35.5% have hypertension, 25.6% have diabetes, 57.9% have bacterial positive sepsis, and 69.4% have MOF. After 90 days, the ratio of AKI to CKD was 22.3%.

There were no differences in sex, hypertension, diabetes, number of WBC, neutrophil, serum PCT, and bacterial positive sepsis between recovery and AKI to CKD patients with *p* > 0.05. However, age, the level of serum urea, creatinine, and the ratio of oliguria and of MOF of AKI to CKD patients were significantly higher than those of recovery ones, *p* < 0.05 (Table 3). Median GFR in recovery patients was higher than the AKI to CKD group, *p* < 0.001. The median concentration of uNGAL and sNGAL (ng/ml) was significantly higher in AKI to CKD patients as compared to the complete recovery patients (sNGAL: 790.99 versus 351.86, uNGAL: 885.72 versus 264.68, *p* < 0.001) (Table 3).

Logistic regression analysis that helps to identify the independent risk factors of AKI to CKD is shown in Table 4. Ages, serum urea, and urine NGAL at the admission were independent risk factors (*p* = 0.004, 0.003, and 0.005, respectively).

**Figure 1:**
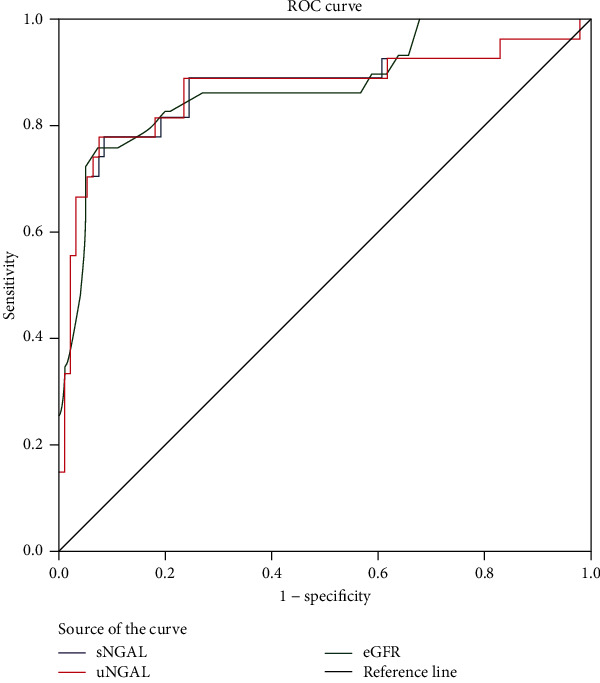
Receiver operating characteristic (ROC) curve of eGFR, serum NGAL, and urine NGAL for prediction of AKI to CKD. eGFR: AUC = 0.857, *p* < 0.001; cut-off value = 18.5 ml/min/1.73m^2^, Se = 74.1%, Sp = 92.6%. sNGAL: AUC = 0.868, *p* < 0.001; cut-off value = 618.72 ng/ml, Se = 77.8%, Spe = 91.5%; uNGAL: AUC = 0.869, *p* < 0.001; cut-off value = 746.97 ng/ml, Se = 77.8%, Spe = 92.6%.

Figure 1 shows the ROC curve for GFR and serum and urine NGAL to predict AKI to CKD patients. All GFR and urine and serum NGAL were good predictors, in which uNGAL was the best one (AUC was 0.869, *p* < 0.001, 77.8%, Spe = 92.6%).

## 4. Discussion

### 4.1. Ratio of AKI to CKD Transition

The ratio of AKI to CKD was 22.3% in our study. To evaluate the progression of AKI to CKD, we focused on SA-AKI patients, excluded other caused AKI patients to reducing confounding factors for our research results. Although a kidney biopsy is the gold standard for assessing kidney tissue damage (especially in patients with AKI who have progressed to CKD), however, a kidney biopsy was not performed (because the patient did not agree) in the study. All study patients were diagnosed CKD after 90 days of follow-up, basing on KDIGO criteria [26]. Until now, there have not been many studies reporting the progression of AKI to CKD in AKI patients, especially in SA-AKI patients. Xu et al. [27] followed 1295 patients with AKI after cardiac surgery for 2 years and detected 6.8% patients from AKI to CKD. The ratio of AKI to CKD in Xu's study was lower than that of our study (6.8% versus 22.3%). Based on the understanding of the pathogenesis of AKI, we found that AKI after cardiac surgery is often associated with contrast and reduced blood flow to the kidneys, while SA-AKI has a complex pathogenesis mechanism. Infections, sepsis, shock, need for mechanical ventilation, and surgery are well recognized as high-risk factors for the development of AKI [28]. Sepsis has been reported to account for approximately 50% of patients with AKI in ICU, and it has been hypothesized that the modulation of proinflammatory cytokines in septic AKI might be beneficial [8, 15]. Sepsis mediated hypoperfusion leading to tubular necrosis traditionally has been cited as the primary pathophysiology for SA-AKI; however, mounting evidence has challenged this paradigm [29, 30]. Numerous drivers for injury now are recognized as playing a role in SA-AKI, including ischemia-reperfusion injury to the glomerulus, inflammation of specific parts of the nephron, hypoxic and/or oxidant stress, cytokine- and chemokine-driven direct tubular injury, and tubular and mesenchymal apoptosis [20, 31, 32]. Sepsis triggers a systemic cytokine and chemokine response. Acute tubular necrosis is driven by both ischemia-reperfusion injury and cytokine-mediated inflammation. Cellular hypoxia is a molecular driver of injury during SA-AKI. Tissue hypoxia in the kidney during sepsis may be defined by inflammation, changes in intrarenal nitric oxide, nitrosative stress or oxygen radical homeostasis, and dysregulation [20, 31, 32]. SA-AKI with the above mechanisms, the kidney will be more damaged than AKI patients after heart surgery.

Recently, the mechanisms underlying AKI to CKD progression involving multiple interactions among injured tubules, immune cells, endothelial cells, and fibroblasts were cleared [33]. Some pathologic mechanisms that persist after AKI described here are similar to those that cause the progression of CKD, suggesting the strong link between AKI and CKD as roles of injured tubules, roles of vascular cells, roles of immune cells, and roles of fibroblasts [33–35].

### 4.2. Predictive Value of AKI to CKD of Serum and Urine NGAL

Comparison of demographic and laboratory characteristics, we knew that age, the level of serum urea, creatinine, and the ratio of oliguria and of MOF in AKI to CKD patients were significantly higher than those of recovery ones, *p* < 0.05 (Table 3). Our results clearly showed the high age, severe kidney damage (expression on increases of serum urea, creatinine levels, ratio of oliguria/anuria, and multiple organ damage) directly related to the poor outcome of SA-AKI (AKI to CKD). We used some characteristics of SA-AKI patients as age, number of WBC, neutrophil cell, level PCT (expression on severe bacterial infection), serum urea level, and GFR to find factors that related independently to AKI to CKD in SA-AKI patients. It was interesting that severe bacterial infection was not related while high age significantly related to AKI to CKD (Table 4). Particularly, high age is an independent factor related to AKI to CKD in SA-AKI patients (Table 4). Several clinical studies have shown that AKI to CKD progression is more likely to occur in the elderly, causes related to nephron loss, inflammaging and immunosenescence, and tertiary lymphoid tissue development [33, 36, 37]. We also found GFR was a good predictor for AKI to CKD transition (AUC = 0.882, *p* < 0.001; Se = 77.8%, Sp = 92.6%) (Figure 1). Reduced eGFR was a consistent strong risk factor in more advanced stages of CKD in Grams et al.'s study [38].

In the study, we found serum and urine NGAL in relationship with the progression of AKI to CKD in SA-AKI patients. The median concentration of sNGAL and uNGAL (ng/ml) was significantly higher in AKI to CKD patients as compared to the complete recovery patients (sNGAL: 790.99 versus 351.86, uNGAL: 885.72 versus 264.68, *p* < 0.001) (Table 3). They were good predictors for the progression of AKI to CKD in SA-AKI patients. NGAL is expressed in a variety of human tissues, including the lung, liver, and kidney, in various pathologic states [39]. The NGAL form is secreted by injured kidney tubule epithelial cells, whereas the dimeric form is the predominant form secreted by neutrophils [40]. NGAL protein expression was detected predominantly in tubule epithelial cells that were undergoing proliferation and regeneration, suggesting a role in the repair process [41]. The urine NGAL is derived predominantly from epithelial cells of the distal nephron, but serum NGAL originates not only from the damaged kidneys. The complications of AKI include increased susceptibility to infections, extrarenal organ damage, the development of CKD, and an increased rate of hospital readmission. NGAL, both in serum and urine, fulfills many of the characteristics important for a useful AKI biomarker and used in early diagnosis, which predicted mortality in not only all-cause AKI but also SA-AKI [11–15]. The results of this study can be simply understood that NGAL indirectly evaluates kidney damage, as NGAL increases mean severe kidney damage. The recovery process in patients with severe kidney damage will be slower, and the rate of transition from AKI to CKD will be higher.

## 5. Conclusion

In conclusion, the ratio of AKI to CKD in SA-AKI-ICU patients was 22.3%. In AKI to CKD patients, the concentration of serum and urine NGAL was significantly higher than that of completely recovered AKI patients. Serum and urine NGAL, measuring at admission time, was a good prognostic biomarker of AKI to CKD in SA-AKI patients.

## Figures and Tables

**Table 1:** Definitions of single organ failure.

Organ	Definition
Liver failure	SOFA ≥ 3 (serum bilirubin ≥ 6 mg/dl) or Maddrey's DF > 32
Renal failure	Defined as AKI, according to the acute kidney injury network criteria
Nervous system failure	SOFA ≥ 3 (Glasgow coma scale ≤ 9)
Respiratory failure	SOFA ≥ 3 (PaO_2_/FiO_2_ < 200 and mechanically ventilated)
Circulatory failure	SOFA ≥ 3 (use of norepinephrine, epinephrine, or dopamine (dopamine > 5 *μ*g/kg/min))
Coagulation failure	SOFA ≥ 3 (platelet count < 50,000/*μ*l)

AKI: acute kidney injury; SOFA: Sequential Organ Failure Assessment.

**Table 2:** Baseline demographic and laboratory characteristics of patients.

Clinical characteristics and laboratory parameters	Mean ± SD/median	*n*, %
Ages (Min-Max)	67.81 ± 16.04 (31 – 91)	N/A
Number of male (*n*, %)	N/A	72 (59.5)
Hypertension (*n*, %)	N/A	43 (35.5)
Diabetes mellitus (*n*, %)	N/A	31 (25.6)
Blood urea (mmol/l)	11 (8.1–18)	N/A
Creatinine (*μ*mol/l)	181 (152–254.5)	

(i) Stage I	N/A	83 (68.6)

(ii) Stage II	N/A	28 (23.1)

(iii) Stage III	N/A	10 (8.3)
eGFR (ml/min/1.73m^2^), median	27 (20–36.5)	N/A
Oliguria (n,%)	N/A	45 (37.2)
WBC (G/l), median	15.03 (12.83–19.59)	N/A

>12 G/l (*n*, %)	N/A	102 (84.3)

<4 G/l (*n*, %)	N/A	19 (15.7)
Neutrophil (G/l), median	12.05 (9.9–15.96)	N/A
PCT (ng/ml)	15.6 (11.2–31.2)	N/A
Na+ (mmol/l)	134.26 ± 7.95	N/A
K+ (mmol/l)	3.9 ± 1.00	N/A
MOF (*n*, %)	N/A	84 (69.4)
Sepsis		

(i) Bacteria positive (*n*, %)	N/A	70 (57.9)

(ii) Bacterial negative (*n*, %)	N/A	51 (42.1)
sNGAL (ng/ml)	415.17 (273.73–613.74)	N/A
uNGAL (ng/ml)	345.08 (138.92–734.59)	N/A
AKI to CKD (*n*, %)	N/A	27 (22.3)

eGFR: estimated glomerular filtration rate; WBC: white blood cell; PCT: procalcitonin; MOF: multiple organ failure; sNGAL: serum neutrophil gelatinase-associated lipocalin; uNGAL: urine neutrophil gelatinase-associated lipocalin; AKI: acute kidney injury; CKD: chronic kidney disease; N/A: not available.

**Table 3:** Comparison of demographic and laboratory characteristics, serum NGAL, urine NGAL in AKI to CKD patients, and completely recovered patients.

Clinical characteristics and laboratory parameters	AKI to CKD patients (*n* = 27)	Recovery patients (*n* = 94)	*p*
Ages	73.41 ± 13.74	66.2 ± 16.36	*0.039*
Number of male (*n*, %)	17 (63)	55 (58.5)	0.678
Hypertension (*n*, %)	11 (40.7)	32 (34)	0.522
Diabetes mellitus (*n*, %)	9 (33.3)	22 (23.4)	0.298
Blood urea (mmol/l)	25.2 (16.7–32.8)	9.55 (7.05–14.12)	*<0.001*
Creatinine (*μ*mol/l)	326 (235–435)	166 (150–199.25)	*<0.001*

(i) Stage I	6 (22.2)	77 (81.9)	*<0.001*
(ii) Stage II	12 (44.4)	16 (17)	
(iii) Stage III	9 (33.3)	1 (1.1)	
eGFR (ml/min/1.73m^2^), median	15 (11–21)	29 (24–39)	*<0.001*
Oliguria (*n*, %)	17 (63)	28 (29.8)	*0.002*
WBC (G/l), median	15.61 (14.2–18.86)	14.91 (12.75–19.74)	0.627

>12 G/l (*n*, %)	25 (92.6)	77 (81.9)	0.179

<4 G/L (*n*, %)	2 (7.4)	77 (18.1)	0.179
Neutrophil (G/l), median	12.31 (10.2–16.12)	11.81 (9.9–16.05)	0.438
PCT (ng/ml)	14.5 (11.2–31.2)	18.35 (11.2–31.2)	0.859
MOF (*n*, %)	23 (85.2)	61 (64.9)	*0.044*

Sepsis			
(i) Bacteria positive (*n*, %)	17 (63) 10 (37)	53 (56.4) 41 (43.6)	0.542
(ii) Bacterial negative (*n*, %)			
sNGAL (ng/ml)	790.99 (623.03–918.38)	351.86 (262.36–497.86)	*<0.001*
uNGAL (ng/ml)	885.72 (747.59–1026.51)	264.68 (128.99–499.02)	*<0.001*

AKI: acute kidney injury; CKD: chronic kidney disease; eGFR: estimated glomerular filtration rate; WBC: white blood cell; PCT: procalcitonin; MOF: multiple organ failure; sNGAL: serum neutrophil gelatinase-associated lipocalin; uNGAL: urine neutrophil gelatinase-associated lipocalin.

**Table 4:** Univariate logistic regression analysis between AKI to CKD and clinical variables in studied patients.

Variable	Adjusted hazard ratio	95% CI	*p*
Ages	1.145	1.044–1.256	*0.004*
Blood urea (mmol/l)	1.186	1.061–1.325	*0.003*
WBC > 12 G/l	30.817	0.762–1246.22	0.069
Neutrophil (G/l), median	0.878	0.751–1.026	0.102
eGFR (ml/min/1.73m^2^)	1.107	0.941–1.302	0.22
uNGAL (ng/ml)	1.009	1.003–1.015	*0.005*

AKI: acute kidney injury; CKD: chronic kidney disease; WBC: white blood cell; eGFR: estimated glomerular filtration rate; uNGAL: urine neutrophil gelatinase-associated lipocalin.

## Data Availability

The data used to support the findings of this study are available from the corresponding author upon request.
